# β-alanine supplementation improves isometric endurance of the knee extensor muscles

**DOI:** 10.1186/1550-2783-9-26

**Published:** 2012-06-14

**Authors:** Craig Sale, Chester A Hill, James Ponte, Roger C Harris

**Affiliations:** 1 Biomedical, Life and Health Sciences Research Centre, Nottingham Trent University, Clifton Lane, Nottingham, NG11 8NS, UK; 2 Southern Cycle Coaching, Fareham, Hampshire, UK; 3 University of Chichester, Chichester, West Sussex, PO19 6PE, UK; 4 Junipa Ltd, Newmarket, Suffolk, UK

**Keywords:** β-alanine, Carnosine, Isometric endurance, Intramuscular buffering

## Abstract

**Background:**

We examined the effect of four weeks of β-alanine supplementation on isometric endurance of the knee extensors at 45% maximal voluntary isometric contraction (MVIC).

**Methods:**

Thirteen males (age 23 ± 6 y; height 1.80 ± 0.05 m; body mass 81.0 ± 10.5 kg), matched for pre-supplementation isometric endurance, were allocated to either a placebo (n = 6) or β-alanine (n = 7; 6.4 g·d^-1^ over 4 weeks) supplementation group. Participants completed an isometric knee extension test (IKET) to fatigue, at an intensity of 45% MVIC, before and after supplementation. In addition, two habituation tests were completed in the week prior to the pre-supplementation test and a further practice test was completed in the week prior to the post-supplementation test. MVIC force, IKET hold-time, and impulse generated were recorded.

**Results:**

IKET hold-time increased by 9.7 ± 9.4 s (13.2%) and impulse by 3.7 ± 1.3 kN·s^-1^ (13.9%) following β-alanine supplementation. These changes were significantly greater than those in the placebo group (IKET: *t*_(11)_ = 2.9, *p* ≤0.05; impulse: *t*_(11)_ = 3.1, *p* ≤ 0.05). There were no significant changes in MVIC force in either group.

**Conclusion:**

Four weeks of β-alanine supplementation at 6.4 g·d^-1^ improved endurance capacity of the knee extensors at 45% MVIC, which most likely results from improved pH regulation within the muscle cell as a result of elevated muscle carnosine levels.

## Background

High-intensity exercise is associated with the formation and net accumulation of carboxylic acid groups, principally in the form of the lactate anion (Lac^-^). Lac^-^production accounts for the generation of 94% of the hydrogen cation (H^+^) concentration in skeletal muscle [1]. Accumulation of H^+^, as a result of high-intensity exercise, may lead to a decline in intracellular pH from around 7.0 at rest [2] to as low as 6.0 [3]. H^+^ accumulation may contribute to fatigue by interfering with several metabolic processes affecting force production [4]. More specifically, the accumulation of H^+^ in skeletal muscle disrupts the recovery of phosphorylcreatine [5] and its role as a temporal buffer of ADP accumulation [6,7], inhibits glycolysis [8] and disrupts functioning of the muscle contractile machinery [9,10].

The extent of the decrease in intracellular pH with the production of H^+^ during exercise is mediated by intramuscular buffers and secondarily by H^+^ transport from muscle. Physicochemical buffers need to be present in high concentrations in the muscle and also require a pKa that is within the exercise-induced pH transit range. Carnosine (β-alanyl-L-histidine) is a cytoplasmic dipeptide found in high concentrations in skeletal muscle [11] and has a pKa of 6.83 for the imidazole ring, which makes it a suitable buffer over the physiological pH range [12,13]. Carnosine is formed by bonding histidine and β-alanine in a reaction catalysed by carnosine synthase, although, in humans, formation of carnosine in the skeletal muscle is limited by the availability of β-alanine [14].

Data from a recent meta-analysis [15] provides support for the assertion that the main mechanism supporting an effect of increased muscle carnosine on exercise performance and capacity is through an increase in intramuscular buffering capacity. Other studies also provide some indirect evidence to support this role [16,17], although this is by no means the only purported physiological role for carnosine that could influence exercise performance and capacity (for review see [18]).

Despite the role played by intramuscular buffers, pH will still fall concomitant with Lac^-^ accumulation. As a result, it is vital to transport H^+^ and Lac^-^ out of the muscle cell to prevent further reductions in intracellular pH, to reduce cellular concentrations of Lac^-^ and allow extracellular buffers to assist in acid–base regulation. During dynamic exercise, transport of H^+^ out of the muscle cell provides the main control over intracellular pH, although physicochemical buffers and, to a lesser extent, metabolic buffers provide the first line of defence. However, under conditions where muscle blood flow is occluded, physicochemical buffers provide the only defence against local changes in pH.

As such, an isometric exercise model provides a suitable means to further test the hypothesis that an increase in muscle carnosine content, following supplementation with β-alanine, improves exercise capacity and performance as a result of enhanced muscle buffering. Isometric contractions at around 15-20% of maximal voluntary isometric contraction (MVIC) can result in increased intramuscular pressures that are sufficient to reduce muscle blood flow [19,20]. However, muscle blood flow is stopped completely at higher intensities [19,20], with the result that the muscle acts as a closed system and the active muscle fibres are solely dependent upon anaerobic energy provision [21]. Isometric endurance hold time is dependent upon the intensity of the muscle contraction with higher percentages of MVIC causing shorter hold times [22]. At fatigue, the maximal accumulation of Lac^-^ in the knee extensor muscles, and therefore decrement in muscle pH, is caused by a moderate rate of lactate production (~1.1 mmol·kg^-1^ dm·s^-1^) accumulated over a moderate time period. The optimal exercise intensity to accumulate lactate is around 45% of MVIC [23]. The Rohmert equation [22] predicts that a constant isometric contraction of the knee extensors will fail to maintain 45% MVIC after approximately 78 s [24].

Therefore, we aimed to examine the effect of β-alanine supplementation on isometric endurance of the knee extensor muscles at 45% of MVIC. Our hypothesis was that isometric hold times at 45% MVIC would be 78 s before supplementation and that these hold times would be increased with β-alanine but not with placebo.

## Method

### Participants

Sixteen physically active males volunteered and were split into a β-alanine and a placebo group. However, 3 participants dropped out of the study (2 from the placebo group and 1 from the β-alanine group) due to sports related injuries sustained during the period of supplementation. As a result, only thirteen participants completed both trials with 6 and 7 being supplemented with placebo and β-alanine, respectively (Table 1). All participants were considered healthy according to a health screening questionnaire and the health screening procedure was repeated prior to each laboratory visit to ensure the health status of the participants had not changed. Participants had not taken any supplement in the 3 months prior to the study and had not supplemented with β-alanine for at least 6 months. Participants were also requested to maintain similar levels of physical activity and dietary intake for the duration of the study and compliance with this request was verbally confirmed with participants prior to commencement of the study. None of the participants were vegetarian and would have consumed small amounts of β-alanine in their diet, typically 50 to 400 mg per day. The study was approved by the institutions Ethical Advisory Committee and all participants provided informed consent.

**Table 1:** Participant characteristics

		**Age (y)**	**Height (m)**	**Body Mass (kg)**	
				**Week 0**	**Week 4**
**β-alanine**	**Mean**	24	1.81	81.6	81.9
**n = 7**	**SD**	7	0.04	10.9	10.8
**Placebo**	**Mean**	21	1.79	80.3	80.1
**n = 6**	**SD**	4	0.06	10.9	11.2

### Experimental design

Participants completed a total of five isometric knee extension tests (IKET), involving chiefly the *m. quadriceps femoris*, with the largest contribution to force production coming from the *m. vastus lateralis*, to fatigue at an intensity of 45% of MVIC force. Two habituation tests (the coefficient of variation [CV] between the two habituation tests was 5.5% for impulse and 7.0% for isometric hold time) were completed in the week prior to the pre-supplementation test. A further test was performed during week 4 as a practice post-test, with the post-supplementation test being performed at the end of the 4 week supplementation period. Testing sessions were separated by a minimum of 72 h and participants were instructed not to perform any vigorous exercise in the 48 h prior to each session.

Participants were supplemented with either 6.4 g·d^-1^ β-alanine (CarnoSyn™, NAI, USA) or an equivalent amount of placebo (maltodextrin; NAI, USA). Participants were first matched in to pairs based upon their pre-supplementation isometric endurance. The β-alanine dosing regimen consisted 800 mg tablets eight times per day at 2 hour intervals or the same regimen for placebo (maltodextrin) tablets. Participants completed a supplementation log to verify compliance, with the degree of compliance being reported at >95% in both groups. Supplementation with β-alanine at this level has consistently been shown to increase muscle carnosine concentrations by around 60% [14,16], with others reporting no non-responders to β-alanine supplementation [16,25,26]. Overall increases have been shown to be between 40% and 80% depending upon dose (between 3.2 and 6.4 g·d^-1^) and duration of administration (between 4 and 10 weeks).

### Experimental procedure

Upon entering the laboratory, participants were secured in an isometric chair in the sitting position with the back-to-thigh angle and the thigh-to-lower leg angle both set at 90°. All tests were conducted using the non-dominant leg. Force output was measured by a strain gauge attached to the lower leg of the participant just above the ankle with kevlar webbing. The strain gauge was attached to a Powerlab/400 system (ADI instruments, UK) connected to a personal computer.

Participants were initially requested to perform three MVICs of the knee extensor muscles separated by 90 s recovery. Participants were then requested to begin the IKET, which involved holding 45% of their highest MVIC force until volitional exhaustion. Participants were deemed to have started the IKET once force output had reached 95% of the target force output for more than 1 s. Fatigue was quantified as the point at which the participants force output fell below 95% of the target force for more than 1 s. The frequency output of the strain gauge was amplified and quantified by the Powerlab/400 and converted to an instantaneous, visual representation of the force output on a computer screen. Participants were required to maintain force output, as close as possible, to the target force, which was indicated by a line superimposed upon the computer screen. In addition to this visual representation, participants were also given verbal feedback when their force output was “too high”, “too low” or “on the line”.

The time the contraction was held above 95% of target force (s) was recorded. From the force output data, the average force and CV about the average force were calculated. The impulse (kN·s) was taken as the product of the average force (N) and the duration of the contraction that was held above 95% of the target force (s). As force output was not controlled at exact levels during the IKET, with some variation possible in relation to the maintenance of the target force by the participant, we calculated the average force over a set time period, determined by the shortest hold time of either the pre- or post-supplementation IKET. The average force of the longer IKET was then calculated up to the time of the shorter IKET. This produced two impulse scores based upon the same time duration, which provided a means of assessing whether changes in average force may have resulted in an increase or reduction in the endurance time held. Importantly, the change in impulse (representative of average force in this case, since the time was the same) from pre- to post-supplementation in the β-alanine group (+0.14 ± 0.58 kN·s^-1^) was not significantly different from the change shown in the placebo group (−0.13 ± 0.58 kN·s^-1^).

### Statistical methods

All data are presented as mean ± 1SD, with statistical significance accepted at *p *≤ 0.05. To examine differences between the two treatment groups, delta values were calculated for each participant for all variables. Independent samples *t*-tests were used to assess differences in all variables between the two treatment groups. This was apart from comparing the actual endurance hold times to those predicted by the Rohmert equation [22] at 0 and 4 weeks. For this, a 3 way mixed model ANOVA was used: (actual hold time (independent measure) x predicted hold time (independent measure) x time (repeated measure)). CV and 95% confidence limits were used to quantify the variability of dependent measures of the placebo group.

## Results

### MVIC force and IKET

Participants were instructed to hold the same absolute force output during the pre- and post-supplementation tests (45% of pre-supplementation MVIC force). Delta values for the β-alanine group (+0.3 ± 1.0%), were not significantly different from the placebo group (−0.1 ± 1.4%). Fluctuations in force held during the 45% MVIC test were assessed by calculating the CV about the mean force held. In both the pre- and post-supplementation tests for both groups the CV was 3.9%, with no significant differences between the two supplementation groups.

**Figure 1:**
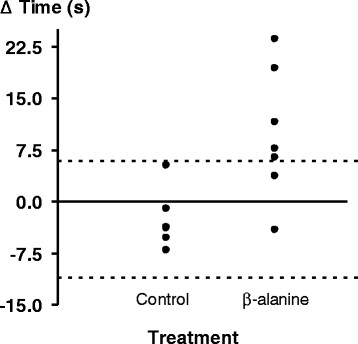
**Vertical line plot of individual participant delta IKET hold-times in the placebo and β-alanine groups.** The horizontal dashed lines represent the ± 95% confidence limits of the placebo group.

IKET hold times, pre- and post-supplementation are shown in Table 2. The 9.7 ± 9.4 s gain (+13.2%) in the β-alanine group was significantly higher (*t*_(11)_ = 2.9, *p* < 0.05) than the corresponding change in the placebo group (−2.6 ± 4.3 s). When examining the individual data (Figure 1), six out of seven participants showed improvements with β-alanine supplementation. When compared to the typical variance associated with the placebo group, five out of seven β-alanine supplemented participants showed improvements greater than the +95% confidence limits associated with the placebo group (+5.9 and −11.1 s).

**Table 2:** Mean ± SD of endurance hold times for the β-alanine and placebo groups

		**Pre (s)**	**Post (s)**	**Delta (s)**	**Change (%)**
**β-alanine**	**Mean**	76.9	86.6	9.7*	13.2*
**n = 7**	**SD**	19.5	21.9	9.4	14.3
**Placebo**	**Mean**	75.0	72.5	−2.6	−4.0
**n = 6**	**SD**	16.7	18.5	4.3	6.6

* denotes a statistically significant difference from placebo at p ≤ 0.05.

A premise of the study was that Lac^-^ plus pyruvate accumulation in muscle were greatest when isometric exercise was performed at 45% MVIC, with fatigue occurring after approximately 78 s [24]. Mean pre-supplementation IKET hold-times were within 4 s of those predicted by the Rohmert curve [22] and applied to the *m. quadriceps femoris* by [24]. There were no significant differences between the actual pre-supplementation endurance hold times and those predicted by the Rohmert curve in either the placebo or β-alanine groups.

### Impulse

**Figure 2:**
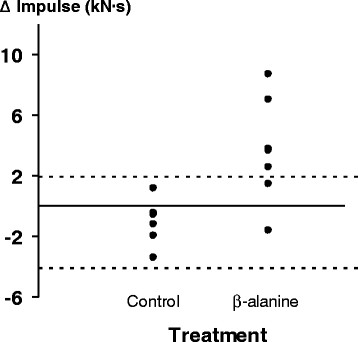
**Vertical line plot of individual participant delta impulse values in the placebo and β-alanine groups.** The horizontal dashed lines represent the ± 95% confidence limits of the placebo group.

We calculated impulse values (IKET hold-time x actual, average force held) to account for participant dependent differences between the force outputs produced pre- and post-supplementation, which might make it a better indicator of performance change than IKET hold-time alone. Impulse values pre- and post-supplementation are shown in Table 3. The 3.7 ± 1.3 kN·s^-1^ gain (+13.9%) in the β-alanine group was significantly different (*t* = _(11)_ 3.1, *p* < 0.05) to the change in the placebo group (−1.1 ± 1.5 kN·s^-1^). When examining the individual data (Figure 2), six out of seven participants showed improvements with β-alanine supplementation. When compared to the typical variance associated with the placebo group, five out of seven β-alanine supplemented participants showed improvements greater than the +95% confidence limits associated with the placebo group (+1.9 and −4.1 kN·s^-1^).

**Table 3:** Mean ± SD of impulse data for the β-alanine and placebo groups

		**Pre (kN·s^-1^)**	**Post (kN·s^-1^)**	**Delta (kN·s^-1^)**	**Change (%)**
**β-alanine**	**Mean**	26.0	29.7	3.7*	13.9*
**n = 7**	**SD**	7.7	9.2	3.4	14.5
**Placebo**	**Mean**	23.4	22.3	−1.1	−4.3
**n = 6**	**SD**	5.6	5.0	1.5	6.1

* denotes a statistically significant difference from placebo at p ≤ 0.05.

## Discussion

In this study we show the effect of 4 weeks of β-alanine supplementation on isometric endurance of the knee extensors at 45% MVIC and demonstrate a 13.2% increase in isometric endurance and a 13.9% increase in impulse. No changes were shown in MVIC force or equalized impulse in either group. As similar average forces were held by participants pre- and post-supplementation, there is evidence that changes in exercise capacity following β-alanine supplementation were related to changes in the capability of the muscle to endure sustained intense isometric exercise. Whilst not the focus of the current study, these results suggest a potential benefit of β-alanine supplementation for several real world applications where isometric exercise is performed (*e.g.*, lifting and carrying, sailing and climbing/mountaineering among other things).

Importantly, endurance hold times for both treatment groups were not significantly different from values predicted by the Rohmert curve [22,24]. The maximal accumulation of lactate and pyruvate, and therefore H^+^ accumulation, is a function of isometric exercise intensity and occurs when MVIC is approximately 45% (when the endurance hold time is around 78 s) [24]. From the data of Ahlborg et al. [24] we estimate that the increase in isometric endurance shown in the β-alanine group would have resulted in the additional accumulation of ~10.7 mmol·kg^-1^ dm Lac^-^ and H^+^ in the muscle. The increase in H^+^ is of the same order as the estimated increase in buffering capacity from the expected increase in muscle carnosine levels, brought about by the programme of β-alanine supplementation (*i.e.*, 6.4 g·d^-1^ β-alanine or 179.2 g in total). From the data of Harris et al. [14] and Hill et al. [16], where participants were supplemented with 145.6 g β-alanine over 4 weeks, we predict that the current supplementation regimen would result in an increase in carnosine in *m. vastus lateralis* of ~18 mmol kg^-1^ dry muscle, an increase of ~70% from an assumed pre-supplementation level of ~25 mmol·kg^-1^ dm. From the Henderson-Hasselbalch equation, which links pKa, pH and metabolite concentration, an increase of 18 mmol kg^-1^ dm would increase buffering by ~9.4 mEq H^+^·kg^-1^ dm over an assumed pH transit range of between 7.1 at rest and ~6.0 at fatigue [3]. Whilst these calculations are a useful way to provide some discussion around the link between H^+^ production and the increase in buffering provided by the elevation in muscle carnosine, it must be noted that this is based upon assumptions relating to the level of increase in muscle carnosine and the exact pH transit range in this study, since muscle biopsy data were not obtained. This highlights a potential limitation of the current study and demonstrates the need for future work to repeat the current study with the addition of mechanistic information provided from muscle determinations of carnosine, Lac^-^ and pH.

Derave et al. [26] previously examined the effects of 4 weeks β-alanine supplementation at 4.8 g·d^-1^ on isometric muscle endurance of the knee extensors at, what was claimed to be, 45% MVIC in trained 400 m runners. In contrast to our results, Derave et al. [26] reported no significant effect of β-alanine on isometric hold-time. However, the pre-treatment times to fatigue reported by Derave et al. [26] were 175 and 201 seconds for the placebo and β-alanine groups, respectively, which brings into question the true intensity of the exercise used in their study given that the hold-time at 45% MVIC would be expected to be ~80s [24]. Using the data of Ahlborg et al. [24], we estimate that the true intensity of the exercise in the Derave et al. [26] study was probably closer to 25% MVIC. At this exercise intensity it is likely that muscle blood flow would have been hampered but that some circulation would have been maintained enabling H^+^ transport from muscle to occur. This would explain the lack of any significant effect of β-alanine supplementation in their study.

The 13.2% increase in IKET hold-time with β-alanine supplementation is comparable with the increases in exercise capacity shown with high intensity cycling following 4 weeks of β-alanine supplementation. In two different studies, increases in exercise capacity were 13.0% [16] and 14.6% [17], providing some evidence of a similar level of effect of β-alanine supplementation on exercise capacity across these studies. There is now increasing evidence to support a positive effect of β-alanine supplementation on high-intensity exercise capacity, mediated through an increase in muscle carnosine, which is further highlighted by a recent meta-analysis of the literature [15].

Whilst a role for carnosine as an intracellular buffer is undisputable, due to both its pKa of 6.83 and its location and concentration in muscle, other physiological roles of carnosine may also contribute to changes in exercise capacity during isometric knee extension exercise. Carnosine has been suggested to increase calcium ion (Ca^2+^) sensitivity in muscle fibres [27,28] and to improve sarcoplasmic reticulum function [29,30], potentially augmenting force production and increasing work done. Both of these effects, however, might also be enhanced by improved pH regulation within the muscle cell [31,32]. Furthermore, neither of these physiological roles for carnosine have been shown in humans and the work cited above has been conducted *in vitro*. Lamont and Miller [28] showed that carnosine reduced the amount of Ca^2+^ required to produce half-maximum tension in chemically skinned cardiac and skeletal muscle and also reported an increase in maximal force production by different muscle types. They suggested that higher concentrations of carnosine, which are shown in fast twitch muscle fibres, might relate to an effect of enhanced Ca^2+^ sensitivity on muscle contractility in fibres capable of producing greater force. Dutka and Lamb [27] showed an increased Ca^2+^ sensitivity of the contractile apparatus, in a concentration-dependent manner, with the addition of carnosine to the cytoplasmic environment. The authors suggested that these results were due to the fact that carnosine sensitises the contractile apparatus to Ca^2+^, without causing additional release from the sarcoplasmic reticulum, thus increasing force production. Whilst the current evidence base for increased Ca^2+^ ion sensitivity in muscle fibres is restricted to *in vitro* work, it would be of interest to examine a possible effect *in vivo*.

The contribution of carnosine to intracellular buffering during isometric exercise might be related to the recruitment pattern of muscle fibres, since different concentrations of carnosine are reported in type I and II fibres [33,34]. Beltman et al. [35] showed that, after seven intermittent 1 s contractions, fibre type activation at 39% MVIC differed between fibres types. Type I and IIa fibres were recruited at 39% MVIC, whereas type IIx fibres were only recruited at 87% MVIC. Progressive shifts in phosphorylcreatine/creatine from low to high percentages of MVIC were greater in type I fibres compared to type IIa fibres, which in turn, were greater than in type IIx fibres, suggesting a progressive activation or rate coding of fibres [35]. However, this study did not examine fibre recruitment in contractions sustained to fatigue by which point, most likely, all fibre types would have been recruited. Of relevance to the issue of fibre involvement, we have previously shown that β-alanine supplementation increases carnosine to an equal extent in both type I and II muscle fibres in *m. vastus lateralis*[16,36].

In conclusion, four weeks of β-alanine supplementation at 6.4 g·d^-1^ improves endurance capacity of the knee extensors at 45% MVIC, which most likely results from improved pH regulation within the muscle cell as a result of elevated muscle carnosine levels.

## Abbreviations

ADP, Adenosine-5'-diphosphate; ANOVA, Analysis of variance; Ca2+, Calcium ion; CV, Coefficient of variation; H+, Hydrogen cation; IKET, Isometric knee extension test; Lac-, Lactate anion; MVIC, Maximal voluntary isometric contraction.

## Competing interests

We declare that we received β-alanine and maltodextrin supplies from NAI to undertake this study, though no additional funding was provided. RCH is retired and an independent paid consultant of NAI but undertook the study whilst the University of Chichester. RCH is named as an inventor on patents held by NAI and first filed, and is in receipt of other research grants for research on β-alanine awarded elsewhere.

## Authors’ contributions

RCH first proposed the study and undertook an initial pilot investigation. All authors were responsible for the development of the final experimental design; CS and RCH were responsible for writing of the manuscript; CAH and RCH were responsible for data analysis; CAH and JP were responsible for data collection; CAH was responsible for reviewing drafts of the manuscript. All authors read and approved the final manuscript.
